# Difference in daily tasks execution and elbow joint load: a comparison between patients after total elbow arthroplasty and healthy controls

**DOI:** 10.1016/j.jseint.2024.10.017

**Published:** 2024-11-27

**Authors:** Roos G.A. Duijn, Daniëlle Meijering, Riemer J.K. Vegter, Alexander L. Boerboom, Denise Eygendaal, Martin Stevens, Claudine J.C. Lamoth, Alessio Murgia

**Affiliations:** a Department of Human Movement Sciences, University of Groningen, University Medical Center Groningen, Groningen, the Netherlands; b Department of Orthopedic Surgery, University of Groningen, University Medical Center Groningen, Groningen, the Netherlands; c Department of Orthopaedics and Sports Medicine, Erasmus University Medical Center Rotterdam, Rotterdam, the Netherlands

**Keywords:** Elbow prosthesis, Elbow joint loading, Activities of daily living, Biomechanical analysis, Varus-valgus, Musculoskeletal modeling, TEA

## Abstract

**Background:**

Overloading of the elbow joint is one of the mechanisms responsible for failure of total elbow arthroplasty (TEA). Different activities of daily living (ADL) affect joint loading. However, the alteration in task execution and its impact on joint loads after TEA are not well understood. This study investigates differences between TEA patients and healthy controls in task execution and associated joint loads during simulated ADL.

**Methods:**

TEA patients (n = 7) and healthy controls (n = 18) performed 8 simulated ADL tasks. Using musculoskeletal modeling software (OpenSim), joint angles and moments were calculated and joint power was assessed. A mixed model statistical design was performed to determine group and tasks differences.

**Results:**

TEA patients showed reduced flexion–extension (FE) range of motion (60.6° ± 25.6 vs. 44.9° ± 19.9, *P* = .003). Interaction effects between groups and tasks for joint load and peak power were observed. Particularly during rising from a chair, patients showed reduced FE moment (5.7 Nm vs. 14.5 Nm, *P* = .026), varus-valgus moment (6.0 Nm vs. 14.3 Nm, *P* = .036), and peak power (3.6 Watt vs. 20.1 Watt, *P* = .036) compared to healthy controls.

**Conclusion:**

TEA patients differ from healthy controls in task execution of ADL tasks regarding the functional elbow FE angle over all 8 ADL tasks and in joint load and peak power for the more straining tasks. The power plots visualizes differences in movement strategy that are of interest for future research on possible training of TEA patients, or prosthesis design, aimed to improve ADL function and enhance prosthesis survival rates.

Total elbow arthroplasty (TEA) is an end-stage treatment option for severe elbow pathologies (eg, rheumatoid arthritis, post-traumatic arthritis, complex distal humerus fractures). Desired outcomes of TEA include decreased pain and restoration of function. Unfortunately, TEA has lower survivorship than other joint replacements,^19^^,^^29^ with 10-year survival rates of around 82%.^35^ The most common reason for revision surgery is aseptic loosening, which is associated with overloading of the elbow joint.^6^^,^^29^ Mechanical overloading of prosthesis occurs when the applied load exceeds the material’s yield strength. Excessive loads could also induce (micro)motions between implant and bone resulting in potential loss of fixation of the implant.^18^^,^^37^

To prevent overloading, the advice after surgery is to lift about 1 kg regularly and incidentally up to 5 kg.^5^ Previous research showed that this instruction does not lead to lower elbow joint loads in healthy adults when performing standardized activities of daily living (ADL) tasks.^7^ Moreover, these instructions are not evidence-based and hard to interpret for patients and clinicians since they are not movement- or task-specific. It is also known that elbow joint load is influenced by how the task is executed and for instance, depends on shoulder position.^33^ As a result, similar movements with similar weights can lead to different joint loads depending on how the task is executed. Therefore, additional insights into the way of execution of tasks by patients with a TEA are necessary to develop postoperative instructions.

It is established that daily task performance improves following TEA, specifically with the restoration of range of motion (ROM).^35^ Welsink et al systematically analyzed the results of the most-performed TEAs, reporting an overall weighted flexion angle of 129° and a weighted mean extension deficit of 30°.^35^ This arc of motion falls within the reported flexion–extension (FE) ROM necessary to execute daily tasks.^28^ Moreover, functional outcomes as ADL performance and pain show improvement after TEA compared to the preoperative state.^19^^,^^29^^,^^30^ These outcomes are often assessed using physician-reported performance scales, such as the Mayo Elbow Performance Scale, which evaluates elbow function in terms of pain, stability, ROM, and daily activities on a range of 1 (poor) to 100 (excellent).^19^ A study by Park et al indicated a clinically relevant increase in the average Mayo Elbow Performance Scale score from 35 preoperatively to 85 postoperatively.^29^

These functional outcome scores do not provide any insights into the task execution or how this influences elbow joint loads and prosthesis wear in the long term. While there is limited knowledge regarding task execution in patients who have undergone TEA and the factors that affect the joint loads acting upon the prosthesis, there are several reasons to expect an altered task execution after TEA. First, the surgical approach (triceps-sparing or triceps-detaching) might lead to altered anatomy and proprioception.^21^ Second, frequently reported complications such as triceps insufficiency and malposition of the prosthetic components could affect task execution. Triceps insufficiency can result from surgical exposure, devascularization, denervation, or intensive rehabilitation.^25^ Reduced triceps strength, leading to a decrease in extension moments, may increase the probability of compensatory movements in other joints, ultimately impacting the performance of daily tasks.^3^ In addition, inadequate positioning of the prosthesis during the surgery might change the joint axis and moment arm of the muscles. These changes lead to altered joint kinematics, possibly causing changes in the task execution.^20^^,^^27^

Biomechanical analysis of task execution during ADL tasks can create more insights into the underlying mechanism of elbow function after TEA. Joint moments and kinematics can be combined to calculate joint power and work.^9^ Joint power (Watt, Joules/s) is the product of joint moment (Nm) and the angular velocity (rad/s) around the joint. Upper-extremity joint power has only been investigated during standardized arm movement or sport-related activities.^8^^,^^10^ Positive joint power indicates that both the movement and the generated joint moment change in the same direction, which indicates a concentric contraction of the muscles. For example, when opening a door, the elbow extensors generate an extension moment during an extension movement, hence there is positive joint work. An opposite moment and movement direction results in negative joint power, indicating an eccentric muscle contraction. Joint work (Joules) is joint power integrated over time. Disbalance of positive and negative joint work within a joint can reveal compensatory movement by other joints.

In previous research, we already found differences in elbow joint moments between different standardized ADL tasks, performed by nonsymptomatic persons and concluded that pushing and pulling tasks, like opening and closing a door are more demanding than lifting a 1-kg object.^7^ The current study considers a group of TEA patients and examines the difference in tasks execution between both groups, in terms of movement time, ROM, joint-moment, joint-power, and joint-work during simulated ADL tasks.

No differences in movement time and FE-ROM are expected since reported FE-ROM outcomes after TEA are within the needed FE-ROM to perform ADL tasks.^28^^,^^35^ Lower joint moments and powers are hypothesized in patients performing ADL tasks due to commonly reported complications such as malposition and triceps insufficiency. We further expect that group differences to be more significant in more demanding tasks indicating task-dependent disparities.

## Materials and methods

### Participants

Patients following TEA (n = 7) and healthy controls (n = 18) participated in the study (Table I). Six of the 18 healthy participants were also enrolled in previous research.^7^ In total, 6 patients with triceps-on approach where included and 1 with triceps flap approach (Table II). The average postoperative time was 2.6 years. (Table II). Inclusion criteria were age 18 years or older, understanding of the Dutch language, and giving informed consent. For the patients’ group, additional criteria were having received a primary TEA (type Latitude evolution) on their dominant hand in the last 1-5 years. Exclusion criteria were mental or physical disability, insufficient command of the Dutch language, prior surgery in the upper extremity or other pathologies affecting upper-extremity function. The study was approved by the Medical Ethical Committee of University Medical Center Groningen, The Netherlands (METc2019/624).

**Table I:** Participant demographics and results of maximum voluntary contraction.

	TEA group (n = 7)	Control group (n = 18)	
	Amount (SD)	Amount (SD)	*P* value∗
Sex (male/female)	2/5	7/11	
Dominance (% right)†	100	89	
Left		−82.5 ± 11	
Right	65 ± 25	87.5 ± 13	
Height (cm)	170.3 ± 10	176.8 ± 13	.236
Weight (kg)	77.7 ± 10	72.8 ± 12	.344
Age (y)	54.7 ± 10	49.4 ± 18	.614
Shoulder width (cm)	43.3 ± 2	45 ± 3	.191
Arm length (cm)	58.7 ± 6	65.2 ± 6	**.017**
Indication‡			
PTA	4		
Gout arthritis	1		
Rheumatoid arthritis	2		
Years postoperation	3.1 ± 1.6	-	
Max extension (deg)§	19.2 ± 14.6	-	
Max flexion (deg)§	121.7 ± 13.7	-	
MVC (N)‖			
Biceps	74.3 ± 26	138.1 ± 41	**<.001**
Triceps	77.1 ± 25	132.3 ± 34	**<.001**

*MVC*, maximum voluntary contraction; *PTA*, post-traumatic arthritis; *TEA*, total elbow arthroplasty; *SD*, standard deviation.

Values are presented as means ± SD.

Bold *P* value indicates significant between groups difference (*P* < .01).

∗ Two-sided *P* value of independent sample t-test.

† Dominance measured with Handiness scale from −100 (left-handed) to 100 (right handed).

‡ Indication for operation of total elbow arthroplasty surgery.

§ Flexion and extension deficit of affected side measured with goniometer in degrees. (0° is full extension, 140° is full flexed).

‖ Maximum Voluntary Contraction, measured with Handheld dynamometer (in newton).

**Table II:** Patients demographics.

Patients	Gender	Age	Length	Weight	Postoperation year	Indication∗	Surgical approach	Max extension	Max flexion	Max pronation	Max supination
01	Male	33	174	65	5	PTA	Triceps-on	35	135	75	35
02	Female	59	164	91	2	Goat arthritis	Triceps-on	10	125	75	75
03	Female	62	163	80	2	PTA	Triceps-on	10	130	90	90
04	Female	62	160	73	3	PTA	Triceps-on	40	110	90	80
05	Female	52	175	78	4	Rheumatoid arthritis	Triceps-on	5	130	90	80
06	Female	62	167	68	1.5	Rheumatoid arthritis	Triceps-on	15	100	80	80
07	Male	53	188	89	1.5	PTA	Triceps flap	0	120	90	70

*PTA*, post-traumatic arthritis.

Note: active range of motion in flexion–extension and pronation-supination direction measured with goniometer in degrees.

∗ Indication for operation of total elbow arthroplasty surgery.

#### Procedures

Primary anthropometric data (length, weight, arm length, shoulder width) and a maximum voluntary contraction (MVC) of the biceps and triceps were collected at entrance (Table I). The MVC measurements were performed using the MicroFET2 Dynamometer (Hoggan Health Industries, Salt Lake City, UT, USA). The dynamometer was programmed to measure the forces in Newton. During the MVC of the biceps, the participant sat on a chair with the knees in 90° flexion and the elbow in 90° flexion. For the triceps MVC, participants adopted the same seated position, with the elbow at 90° flexion and the hand on the table at 0° pronation-supination (PS) (thumb side facing upwards). Active FE and PS ROM were measured for the TEA patients.

After a static calibration trial, participants performed a standardized series of 8 ADL tasks (Table III). Tasks were selected based on frequently performed ADL tasks, the expected amount of elbow movement, and patient’s frequently asked questions after TEA.^1^ In the seated tasks, the participant sat on a height-adjustable chair without back support. A 75-cm high table with a marked starting point was placed in front of the participant. To standardize the beginning position of each participant, the chair’s height was adjusted so that the elbow was flexed at 90° when the hand was placed on the table, and the upper arm was held in a vertical position. Each task was repeated 5 times after verbal instruction and 1 test trial.

**Table III:** Description of activities of daily living.

	Activity	Initial position	Aim
	1 Steering a car wheel (T-bar horizontal on a table).	A table placed in front of the participant, at 1 AL. Chair lowered, legs are stretched. Dominant hand on T-bar, nondominant hand on leg.	Turn T-bar, using the handle, from 10 o’clock till 16 o’clock and turn back to 10 o’clock.
	2 Opening and closing a door (T-bar vertical on table).	A participant stands in front of the door, with an elbow in 90 degrees, hand resting on the handle.	Push the T-bar to 90 degrees using the handle. Close the door, back to the starting position.
	3 Rising from a chair (T-bar in armrest).	Seated, both hands on armrests.	Rise from chair using armrests, sit down again.
	4 Lifting 1-kg object	Target X on platform (SH) placed 1 AL and 1 SW from the dominant arm. Hold 1-kg object at SP.	SP, place object at target X, back to SP.
	5 Sliding 1-kg object	Target X at 1 SW on the nondominant side, 1 AL with the dominant arm. Hold 1-kg object at SP.	SP, slide target to target X, back to SP.
	6 Combing hair	Rest hand on SP.	SP, combing hair in the midline back and forth, SP.
	7 Drinking (1 kg)	Hold 1 kg object at SP.	SP, simulate drinking, SP.
	8 Emptying a cup (1 kg)	Target at 1 full AL in front of the participant. Hold 1-kg object at SP.	SP stretch arm towards target, 180° rotation, counterclockwise, then back, SP.

*AL*, arm length, measured from the dominant acromion to third; *SW*, shoulder weight, measured from acromion to acromion; *SH*, shoulder height; *SP*, starting point.

Each activity was repeated 5 times, all within 35 sec. A T-bar is an aluminum bar implemented in a force transducer.

The participant was instructed to move at a comfortable speed throughout the experiment. There was a rest period of 30 seconds between the different tasks.

#### Instruments and data collection

Body segment position of the upper extremity was collected at 100 Hz using a 4-position sensor motion capture system (Optotrak 320; Northern Digital Inc., Waterloo, ON, Canada). Four infrared light–emitting markers were placed on bony landmarks of the upper limb and thorax. Six rigid bodies were placed on the thorax and upper-limb segments, which mapped 14 additional virtual markers. Last, 1 marker was placed on the center of the force, Z to the right, and Y upward.

**Figure 1:**
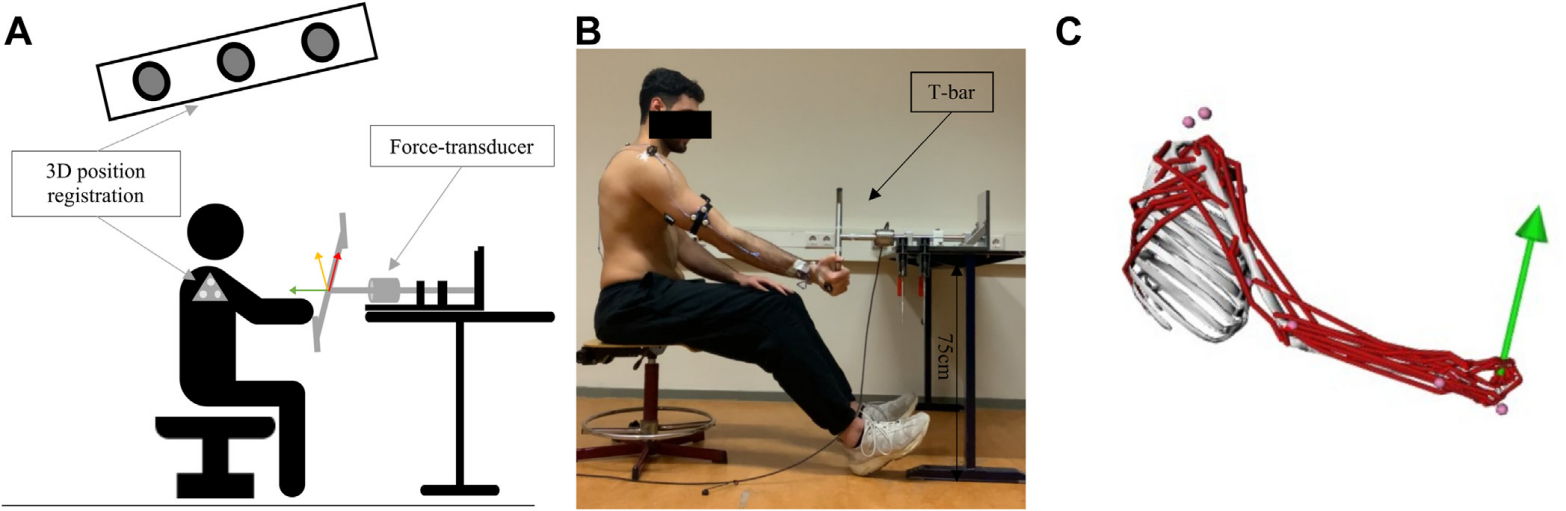
Schematic overview of experimental setup for car task with the force transducer (15-N resistance force). (**A**) Sagittal view of the lab setting. (**B**) View of the geometry of the musculoskeletal model. (**C**) View during the car task. The markers are in pink dots, the muscles in red, and the green arrow represents the external reaction force (force from T-bar to hand).

Force data were recorded with a force transducer (ME-Messysteme GmbH, Henningsdorf, Gtransducer and one marker on the 1-kg object (Fig. 1). All marker positions are shown in Supplementary Appendix A. The coordinate system of the marker data was set on the table, with X forwardermany) with an accuracy of 0.01 N. The force transducer was mounted on an aluminum T-bar and could be set in different positions (Fig. 1*A*/*B*). Both marker and force data were simultaneously measured at 100 Hz.

#### Data analysis

The force values in the local coordinate systems were converted into a global coordinate system of the motion capture system using a customized MATLAB script (version 20a; MathWorks Inc., Natick, MA, USA). The anatomical locations and the segments’ coordinate systems are in accordance with the International Society of Biomechanics recommendations. Data gaps of up to 20 data points were reconstructed using piecewise cubic spline interpolation. Subsequently, a fourth-order Butterworth filter with a 6-Hz cut-off frequency was employed to filter the motion capture and force data.

Inverse kinematic and inverse dynamic analysis was performed in OpenSim musculoskeletal modeling software (version 4.4; Stanford University, Stanford, CA, USA). The dynamic Holzbauer model was scaled in OpenSim to the body dimensions of the participant. This model comprises 7 bone segments and 50 Hill-type muscle-tendon actuators, representing 32 muscles and muscle compartments.^7^ The Holzbauer model was adjusted to include the varus-valgus (VV) moments. For further details, see Duijn et al 2023. The external reaction forces were applied to the model’s hand segment at the distal end of the third metacarpal bone of the dominant hand. The gravitational force was applied to the hand during the task where the 1-kg object was lifted. In the task where a 1-kg object was shifted across the table, a dynamic friction coefficient of 0.5 for polyethylene (PE)-on-polyethylene was applied.

**Figure 2:**
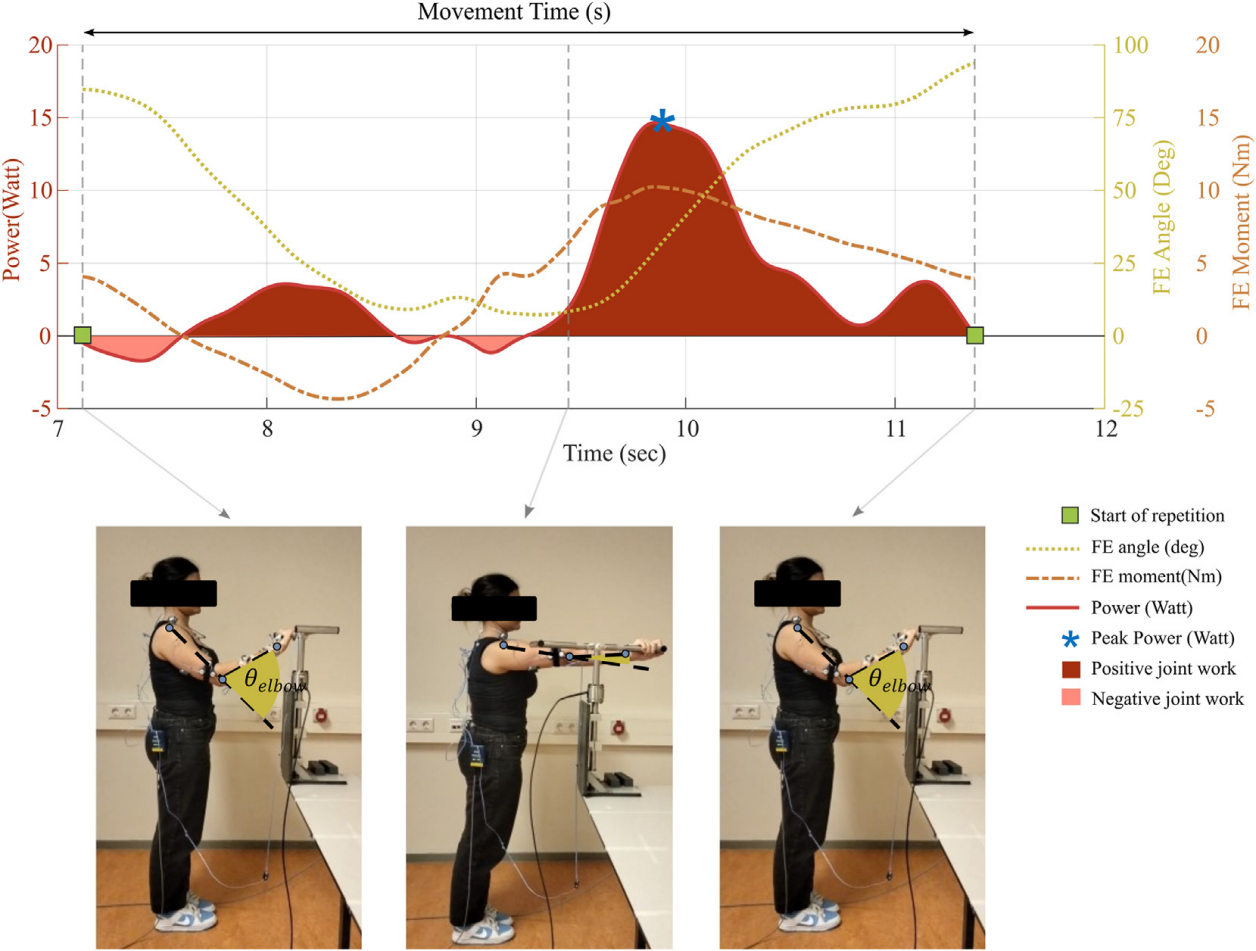
Example of power calculation during door tasks. The movement time is defined as the duration from the start to the end of the tasks. Flexion-extension (FE) angle (θ_angle) is the angle between the lower arm and the extended line from the acromion to the epicondyle. The range of motion is defined as the maximal angle – minimal angle. Joint work (in Joules) is calculated as the integral of power over time. Positive power indicates concentric contraction, while negative power indicates eccentric contraction. *FE*, flexion-extension.

Further data analysis was performed in MATLAB (R2022b). The first and last repetitions were removed. One of the 3 middle repetitions was selected for further analysis based on missing marker and a clear start and end. The start and end of the repetition are determined by the FE angle (Fig. 2). Movement time was calculated as the time between the start and end of 1 repetition. ROM in the FE and PS direction was calculated based on maximum and minimum joint angle. Peak FE and VV moments were calculated based on absolute values during the third repetition (Fig. 2). No PS moments were analyzed, as previous research indicated that the peak FE and VV moments were significantly higher than the peak PS moment and therefore less impact on prosthesis wear.^7^ Angular velocity was calculated based on 3-dimensional kinematics using Euler angles relative to the right-handed coordinate system.^4^ Joint power for the FE direction was computed in MATLAB using angular velocities (ω) and inverse dynamic output ($M_{FE\_elbow}$) (eq 1). Joint work is calculated by integrating the joint power over time (eq 2).

(eq1) $$Joint\;Power\;\left(Watt\right)=M_{FE\_elbow}*\omega$$

(eq2) $$Joint\;Work\;\left(Joules\right)=\int_{t_{1}}^{t_{2}}P_{elbow}\left(t\right)dt$$

#### Statistical analysis

Differences in task execution between TEA patients and healthy controls were determined based on the following outcome variables: 1) movement time, 2) ROM in FE and PS direction (deg), 3) peak joint moment (Nm), 4) peak joint power (Watt), 5) total joint work (Joules). To analyze the data, IBM SPSS Statistics 29 (IBM Corp., Armonk, NY, USA) was used to perform a mixed models design for each variable, with groups (TEA vs. control) as between-group factor and tasks (car, door, chair, etc.) as within-group factor.

The main effect of group was examined to assess differences between TEA patients and healthy controls across all tasks. The interaction effect of group and task was investigated to determine whether group differences were task-dependent. Independent t-test with Bonferroni correction was used as post hoc test. Cohen *d* was calculated to indicate effect size. Since data were not normally distributed, they were normalized via a Z-transformation. A *P* value < .05 was considered statistically significant.

## Results

The mean group values and statistical outcome of movement time, ROM, peak joint moment, peak joint power, and joint work are presented in Table IV. External force data were missing for 1 TEA patient and 1 healthy control; therefore, only movement time and ROM of these participants were analyzed. Significant differences between tasks were observed for all variables.

**Table IV:** Difference in task execution between patients following total elbow arthroplasty and healthy controls.

Outcome	Group	N	Mean (SD)	Group∗	*P*	Task†	*P*	Group∗task†	
				F		F		F	*P*
Movement Time(s)	TEA	7	5.0 ± 1.8	1.610	.217	4.253	**<.001**	.657	.708
	Control	18	4.3 ± 1.4						
FE-ROM (Deg)	TEA	7	49.1 ± 21.6	10.476	**.003**	53.540	**<.001**	1.929	.068
	Control	18	62.2 ± 25						
PS-ROM (Deg)	TEA	7	58.9 ± 36.4	0.68	.797	13.772	**<.001**	.851	.547
	Control	18	57.4 ± 44.4						
FE Moment (Nm)	TEA	6	6.8 ± 2.9	2.357	.138	11.177	**<.001**	4.711	**<.001**
	Control	17	8.0 ± 4.6						
VV moment (Nm)	TEA	6	7.4 ± 4.0	0.359	.555	26.549	**<.001**	4.213	**.001**
	Control	17	7.9 ± 5.6						
Peak power (Watt)	TEA	6	4.6 ± 4.7	2.202	.151	4.372	**<.001**	3.634	**.001**
	Control	17	7.0 ± 9.0						
(Concentric) work + (Joule)	TEA	6	2.9 ± 3.3	0.153	.300	6.533	**<.001**	.988	.074
	Control	17	3.7 ± 4.4						
(Eccentric) work - (Joule)	TEA	6	−2.1 ± 2.4	1.123	.699	7.436	**<.001**	1.891	.442
	Control	17	−1.9 ± 2.0						

*FE*, flexion–extension; *ROM*, range of motion; *PS*, pronation-supination; *VV*, varus-valgus; *SD*, standard deviation.

Tasks execution is defined as movement time, FE-ROM, PS-ROM, peak FE moment, peak VV moment, peak power, and joint work.

Bold values show *P* value <.05.

∗ Degrees of freedom for the numerator were 1 and for the denominator were 23.

† Degrees of freedom for the numerator were 7 and for the denominator were 161.

### Movement time and ROM

**Figure 3:**
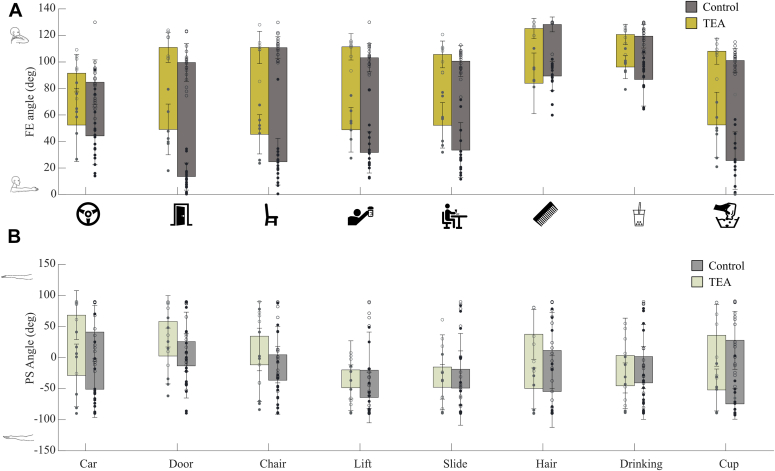
Range of motion of the elbow during 8 daily activities for patients following total elbow arthroplasty and healthy controls. (**A**) The flexion–extension angle is defined from 0° (full extension) to 130° (full flexion). (**B**) The pronation-supination angle is defined from −90° (full pronation to 90° (full supination). Open dots represent the maximal angle, and filled dots represent the minimal angle.

No main group effect was found for movement time (mean TEA 5.0 ± 1.8 seconds and control 4.3 ± 1.4 seconds). Main group effect was found for FE-ROM. TEA patients performed the tasks with a smaller FE-ROM (range 61.5°-110.6°), especially with less extension of the elbow, compared to healthy controls (range 43.4°-105.7°). Fig. 3 illustrates the average ROM of each task in the FE and PS directions. Both groups exhibited the largest FE-ROM during the door, chair, lift, and cup tasks. High PS-ROM was observed during the cup task in both groups.

### Joint moments

**Figure 4:**
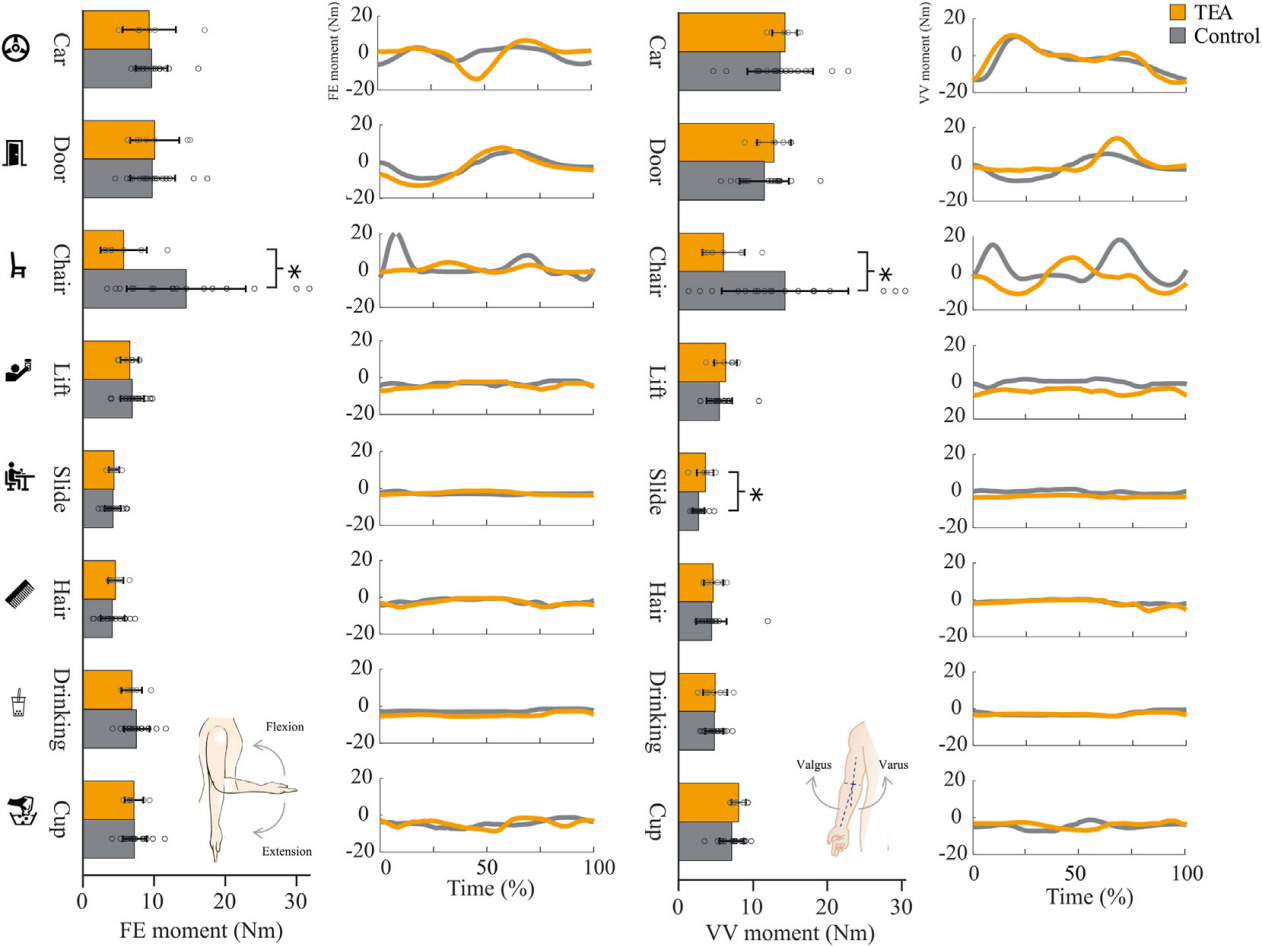
Peak absolute elbow joint moments in the flexion–extension and varus-valgus direction for patients following total elbow arthroplasty and healthy controls. The error bars represent the standard deviation, and the dots represent the individual peak moment. Independent t-test was performed with ∗ indicating *P* < .05. The time series shows the joint moment (in Nm) during the movement, for an average total elbow arthroplasty patient and healthy control, normalized over time.

Fig. 4 shows the joint moments in FE and VV directions. Overall, there was no main group effect in peak FE and VV moments. However, there was an interaction effect between group and task for both FE moment and VV moment (Table IV). Post hoc test revealed that during the chair tasks, TEA patients showed lower FE moment (mean 5.7 ± 4 Nm) compared to healthy controls (mean 14.5 ± 9 Nm) (t (1,23) = 2.92, *P* = .026, Cohen’s *d* = 1.8) and also showed a lower VV moment (t (1,23) = 2.236, *P* = .036, Cohen’s *d* = 1.5). During the slide tasks, TEA patients showed a higher VV moment than healthy controls (t (1,23) = 2131, *P* = .044, Cohen’s *d* = 0.2). Highest joint moments were observed during the door, car, and chair tasks.

### Joint power and joint work

**Figure 5:**
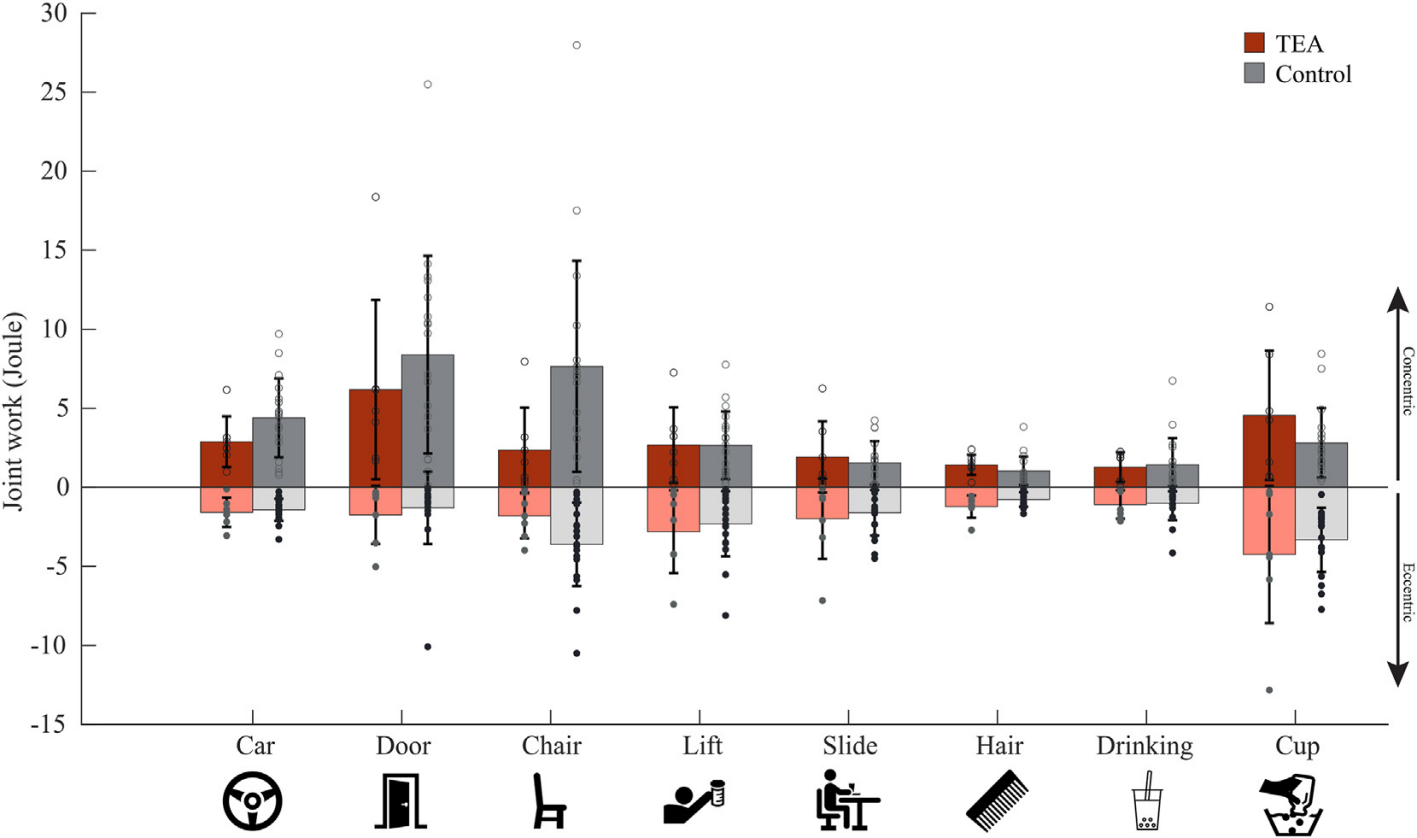
Elbow flexion–extension joint work for 8 daily activities in patients following total elbow arthroplasty and healthy controls. Positive joint work indicates a concentric contraction, and negative joint work indicates an eccentric contraction. No significant difference was found for all tasks.

There was no main group effect for peak power between the TEA group (mean 4.6 ± 4.7 Watt) and control group (mean 7.0± 9 Watt). However, again an interaction effect was observed between group and task. Follow-up analysis showed that only during the chair task, the TEA group showed a significant decrease in peak power (t (1,23) = 2.245, *P* = .036, Cohen’s *d* = 0.2). There was no main group effect for both concentric work (mean TEA 2.9 ± 3.3, control 3.7 ± 4.4) and eccentric joint work (mean TEA −2.1 ± 2.4, control −1.9 ± 2.0). Total concentric (positive) and eccentric (negative) joint work in the FE direction for each task are shown in Fig. 5. The highest concentric amount of joint work was observed during opening and closing a door for the TEA group (mean 6.8 ± 6 Joules) and control group (mean 8.4 ± 5 Joules). The highest eccentric amount of joint work was observed during emptying a cup for the TEA group (mean 4.2 ± 4 Joules) and control group (mean 3.3 ± 2 Joules).

**Figure 6:**
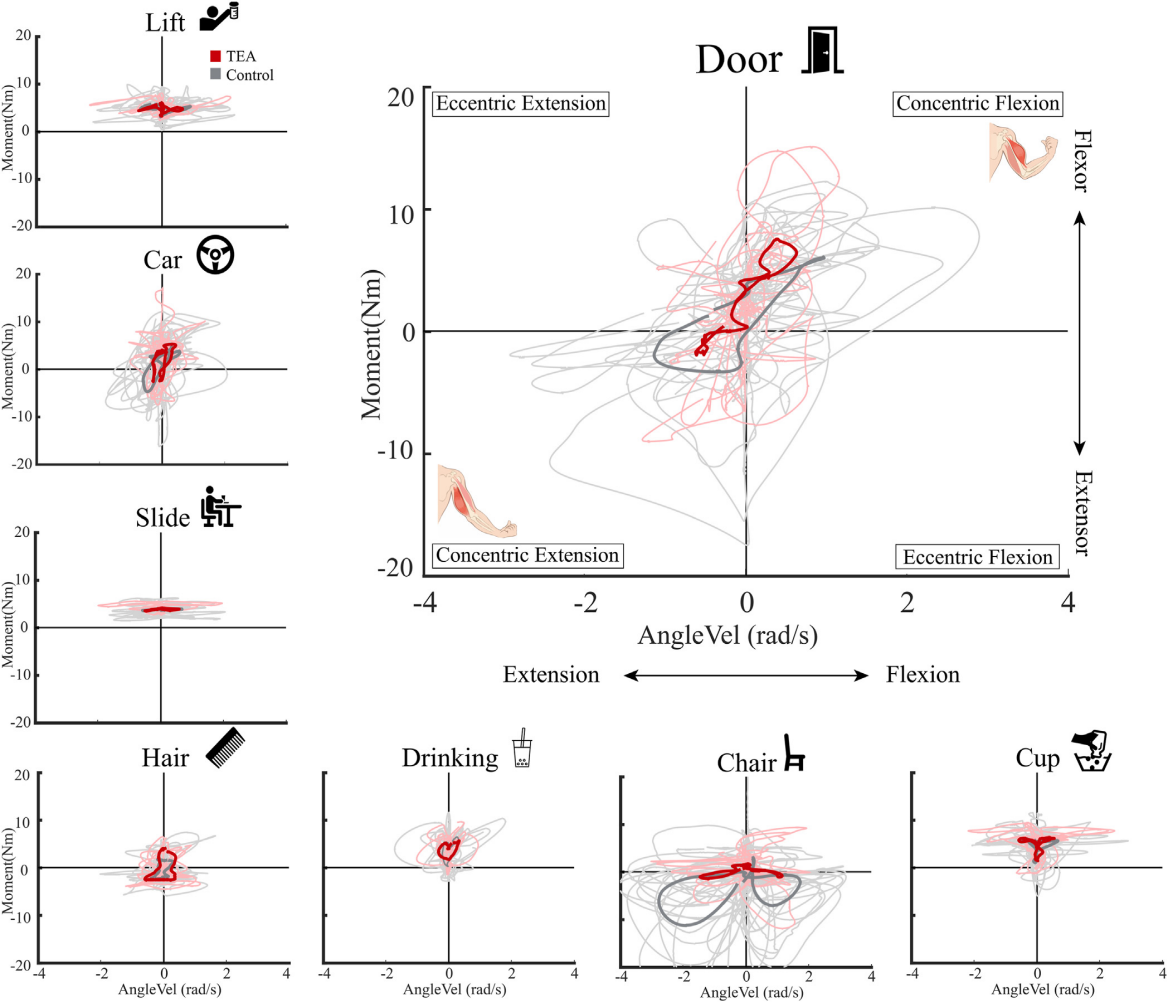
Power plots of 8 daily activities. The pink and light gray lines show the individual trial of each participant. The bold red line shows the mean movement pattern of the TEA group, and the bold gray line shows the mean of the control group. Each quadrant in these plots shows a type of contraction (concentric contraction of the flexors, eccentric contraction of the flexors, concentric extensors, eccentric extensors). A positive moment indicates that the flexor muscles are active, and a negative moment indicates that the extensors are active. A positive angular velocity indicates a flexion movement, and a negative angular velocity indicates an extension movement. *TEA*, total elbow arthroplasty.

Fig. 6 shows the power plots of the 8 ADL tasks. Each quadrant in these plots shows a type of contraction (concentric contraction of the flexors, eccentric contraction of the flexors, concentric extensors, eccentric extensors). The different patterns indicate that each task requires its own movement strategy. The high variability between participants indicate high intrasubject variability. During the chair tasks, the TEA group shows a different movement strategy than the control group; they produce lower moments but the same speed. During the door tasks, the TEA group produced the same amount of joint moment as the controls but with less speed.

## Discussion

The current study examined differences in daily task execution between TEA patients and healthy controls. Task execution was quantified using movement time, ROM, joint-moment, power, and work. No group effect for movement time was found. Contrary to our expectations, a group effect was found for the FE-ROM between TEA patients and controls, with TEA patients showing a reduced elbow extension angle over all tasks. The anticipated lower joint moments and peak powers for the TEA group were not observed as a main group effect, but were shown to be task-dependent, as indicated by the group and task interaction effect. Particularly in the most power-demanding task, rising from a chair, TEA patients exhibited lower joint moments and peak power than healthy controls. No group effect was found for joint work. Despite performing the same tasks with the same instructions as the healthy controls, TEA patients showed differences in task execution as illustrated by the power plots.

### Movement time and ROM

No differences in movement time were found. Exploring the impact of movement speed on joint loads remains interesting, as slower movements may compensate for the differences in peak power output by spreading the same amount of work over a longer time period.^13^ TEA patients exhibited reduced elbow extension angles during the 8 ADL tasks compared to the healthy controls. An important factor is that the patient group was found to have an extension deficit. However, despite this deficit, they were physically capable of attaining a similar ROM to that of the healthy controls. For instance, patients exhibited a minimal FE angle of 48.8° during the lifting task, whereas healthy controls displayed an average minimal angle of 31.7°. It is essential here that 6 of the 7 patients could reach the 31.7° FE angle, but these angles were not observed during this task due to movement strategy. Several explanations exist. First, there could be a lack of proprioception in patients following TEA, as reported by another study^32^ which could lead to altered FE angles. Secondly, the different movement strategies could have resulted from the lower muscle strength that patients have compared to healthy control, as shown in Table I. Lastly, a possible explanation could be that TEA patients used a different movement strategy as a protective mechanism, causing them to perform tasks more cautiously. Due to painful motion, this protective mechanism might already exist years before surgery. From a patient’s perspective, the difference in FE-ROM may cause compensatory movements in other joints, requiring relatively higher muscle forces. This increased effort could result in earlier onset of fatigue. However, further research is needed to confirm these findings.

More interesting is to see if the altered FE angles were accompanied by lower joint loads. Previous research indicated that peak elbow joint loads are amplified when the arm is fully extended.^2^^,^^15^^,^^36^ From a biomechanical perspective, it can be observed that the moment arm generally shows greater values during arm extension than in a flexed position closer to the body. Furthermore, muscle activity is higher early in the flexion cycle due to the poor mechanical advantage of the prime movers: the m. brachialis, m. biceps brachii, and m. brachioradialis. Higher elbow joint loads would accompany higher muscle activity with an outstretched arm.^15^ Consequently, TEA patients may experience reduced joint loads by limiting the full extension of the elbow joint.

### Joint loads

The anticipated lower-joint moment and power values for the TEA group were not observed as a main group effect, but were shown to be task-dependent, as indicated by the group and task interaction effect. The most notable differences occurred when rising from a chair, where the TEA group showed significantly lower FE and VV moments. After TEA, patients receive postoperative instructions from their physicians, advising them not to lift more than 1 kg.^5^ Previous findings suggest that this instruction may impact the elbow joint loads when rising from a chair. Patients can adapt and compensate for this movement through their legs,^7^ which might explain the lower loads.

TEA patients also exhibited higher VV moments during the slide tasks than healthy controls. Higher VV moments could potentially result from compensatory movements originating from the shoulder.^16^ Although we did not measure the shoulder abduction angle in our study, it was kept standardized for most tasks (see Table III). Besides shoulder abduction, axial rotation of the shoulder could also influence the VV loads. When the shoulder is at 90° and moving in the plane of elevation, the amount of axial rotation of the humerus can influence the stresses on the collateral ligaments, ultimately affecting the VV moments.^14^

Furthermore, it is important to emphasize that the peak VV moments in TEA patients during 6 tasks exceeded 5 Nm. VV moments above this value were shown by Lo and Lipman to exceed the yield strength of PE in the Coonrad/Morrey prosthesis, leading to irreversible PE deformation.^22^ However, the TEA patients in our study had a Latitude evolution elbow prosthesis of which the failure limits has not yet been studied in the context of daily tasks.

### Joint power and joint work

The differences in power plot patterns reveal task specific differences between TEA patients and healthy controls, again, especially in the more straining task. The most notable differences in task execution among groups were observed in the door and chair tasks, which allowed for more variability in execution, potentially resulting in compensatory movements in the shoulder, trunk, or legs, as also reflected in joint work results. The positive (concentric) and negative (eccentric) joint work were unequal. Since the total energy is expected to be conserved in a cyclic task, an imbalance indicates that other joints also contribute to the energy (in the form of joint work) necessary to complete the tasks.

The results of the cup tasks indicate that TEA patients showed higher joint work compared to the controls. This trend could be explained by the longer movement time required for the cup task in the TEA group, potentially increasing overall joint work. However, this raises questions about which task execution—long, slow movements or short, quick movements—leads to more prosthesis wear. An in vitro loading study of the cement–bone interface in knee prosthesis suggests that if the loads on the prosthesis stay below the failure limit, the risk of fixation loss, and therefore prosthesis wear, could be limited.^24^ Slowing movement to keep the loads below the failure limits could result in less wear. However, more research is needed on the effect of movement speed and prosthesis wear.

### Future recommendations

The present study employed a generic musculoskeletal model derived from a healthy male subject.^12^ While this estimation proves adequate for calculating net joint moments and joint angles within our study,^11^ we advocate that future investigations should employ personalized musculoskeletal models. This recommendation is particularly important considering that TEA is more commonly performed in women than in men.^31^ These models constructed using, for example, CT scans, can be tailored to individual characteristics such as restricted FE-ROM, VV laxity, prosthetic integration, and sex differences in anthropometric data.^17^^,^^26^ These advancements will also allow for more precise estimation of joint contact forces in future research and facilitate a more nuanced exploration of the impacts of specific surgical interventions, such as triceps reattachment and FE axis alignment, on elbow joint loads.^23^^,^^30^^,^^34^

As previously shown, generic instructions like “do not lift more than 1 kg” are ineffective in reducing elbow joint loads.^7^ We propose 2 key strategies for formulating postoperative loading instructions to address this. Firstly, understanding the relationship between task execution and elbow joint loads is important. Based on the current results, we recommend investigating the impact of movement speed, shoulder movements, and trunk flexion on elbow joint load. Instead of group comparison, we advise investigating individual movement patterns and recognizing the substantial variability observed in task execution among TEA patients and healthy individuals, as shown in the current results. Secondly, the failure limits of the prosthesis material need to be investigated in both FE and VV directions using finite element analysis.^23^ This will enable the comparison of the measured in vivo elbow loads with the prosthesis limits. To make this comparison, it is crucial that elbow loading is not only calculated based on the external total moments but also on the internal joint contact forces.

## Conclusion

TEA patients differ from healthy controls in task execution of ADL tasks regarding the functional elbow FE angle over all 8 ADL tasks and in joint load and peak power for the more straining tasks. The power plots visualize differences in movement strategy that are of interest for future research on possible training of TEA patients, or prosthesis design, aimed to improve ADL function and enhance prosthesis survival rates.

Based on these findings, future research should further explore internal joint contact forces and the influence of movement speed and shoulder movements on elbow loads. This study serves as an initial step in identifying crucial aspects of task execution that play a key role in alleviating elbow loads following TEA to increase survival rates.

## Acknowledgments

The authors thank Roy E. Stewar for the statistical support and the Technical Support Department of Human Movement Science. They also thank the following people for contribution to measurements: Ronja Schuitema, Maureen Poelstra, Isabel Gilde, Ralph Chewan, Ella Rizk, Pim Beute, and Cas Douwstra.

## Disclaimers:

Funding: No funding was disclosed by the authors.

Conflicts of interest: The authors, their immediate families, and any research foundations with which they are affiliated have not received any financial payments or other benefits from any commercial entity related to the subject of this article.
