# Supplementary material for: Difference in daily tasks execution and elbow joint load: a comparison between patients after total elbow arthroplasty and healthy controls
Source: JSES Int. 2024 Nov 27;9(2):580–9. doi: 10.1016/j.jseint.2024.10.017 (PMC11962619; doi:10.1016/j.jseint.2024.10.017)
Supplement: Supplementary Appendix [file mmc1.docx]

**APPENDIX**

Appendix A: Marker position for 3D marker tracking.

| **Type** | **Segment** | **Location on body** |
| --- | --- | --- |
| **Rigid body** | Sternum | Jugular incision |
| Virtual marker | Xiphoid process | Xiphoid process |
| Virtual marker | Clavicle | Incisura jugularis |
| Single body + virtual marker | C7 vertebra | Spinal process of C7 vertebra |
| Single body + virtual marker | Non-dominant shoulder | Acromion |
| **Rigid body** | Right upper arm | Lateral upper right arm, 1/4 on the line between acromion and lateral epicondyle of humerus |
| Virtual marker | Right elbow lateral | Lateral epicondyle of humerus |
| Virtual marker | Right elbow medial | Medial epicondyle of humerus |
| **Rigid body** | Right forearm | Lower lateral surface of right forearm, one finger width proximal of styloid process of the radius and ulna |
| Single body + virtual marker | Right wrist ulnar | Styloid process of ulna (opposite each other!) |
| Single body + virtual marker | Right wrist radial | Styloid process of radius |
| **Rigid body + virtual marker** | 3^rd^ MCP of finger | 3^rd^ MCP of hand |
| Virtual marker | 5^th^ MCP of finger | 5^th^ MCP of hand |
| **Rigid body** | Sacrum | Sacrum |
| Virtual marker | Right posterior superior iliac | Right posterior superior spine of ilium |
| Virtual marker | Left posterior superior iliac | Left posterior superior spine of ilium |
| Virtual marker | Right anterior superior iliac | Right anterior superior spine of ilium |
| Virtual marker | Left anterior superior iliac | Left anterior superior spine of ilium |
| Virtual marker | T10 vertebra | Spinal process of T10 vertebra |
| **Rigid body + virtual marker** | Right shoulder | Dominant acromion |
| virtual marker | Right shoulder | Acromion angle |
| virtual marker | Right shoulder | Inferior angle |
| virtual marker | Right shoulder | Trigonum spinae |
| virtual marker | Right shoulder | Coracoid process |
| virtual marker | Right shoulder | AC most dorsal point |
|  | 1-kg object – Force transducer | Center of rotation (UP steering, SIDE door) |

Appendix B: Patiënts demographics

| Patients | Gender | Age | Length | Weight | Year post operation | Indication^*^ | Surgical approach | Max extension | Max flexion | Max pronation | Max  supination |
| --- | --- | --- | --- | --- | --- | --- | --- | --- | --- | --- | --- |
| 01 | Male | 33 | 174 | 65 | 5 | PTA | Triceps-on | 35 | 135 | 75 | 35 |
| 02 | Female | 59 | 164 | 91 | 2 | Goat arthritis | Triceps-on | 10 | 125 | 75 | 75 |
| 03 | Female | 62 | 163 | 80 | 2 | PTA | Triceps-on | 10 | 130 | 90 | 90 |
| 04 | Female | 62 | 160 | 73 | 3 | PTA | Triceps-on | 40 | 110 | 90 | 80 |
| 05 | Female | 52 | 175 | 78 | 4 | Rheumatoid arthritis | Triceps-on | 5 | 130 | 90 | 80 |
| 06 | Female | 62 | 167 | 68 | 1.5 | Rheumatoid arthritis | Triceps-on | 15 | 100 | 80 | 80 |
| 07 | Male | 53 | 188 | 89 | 1.5 | PTA | Triceps-flap | 0 | 120 | 90 | 70 |

*Indication for operation of total elbow arthroplasty surgery. PTA = post traumatic arthritis. *Note:* active range of motion in flexion-extension and pronation-supination direction measured with goniometer in degrees

Appendix C: Outcomes of daily task execution parameters for individual tasks

|  |  |  | Total | Tasks |  |  |  |  |  |  |  |
| --- | --- | --- | --- | --- | --- | --- | --- | --- | --- | --- | --- |
| Outcome | Group | N | Mean (SD) | Car | Door | Chair | Lift | Slide | Hair | Drinking | Cup |
| Movement Time (s | TEA | 7 | 5.0± 1.8 | 5.1 (2) | 5.0 (1) | 5.1 (1) | 4.7 (2) | 4.6 (2) | 5.1 (3) | 4.4 (2) | 5.9 (2) |
|  | Control | 18 | 4.3 ± 1.4 | 4.2 (1) | 4.0 (1) | 4.4 (1) | 4.7 (1) | 3.9 (1) | 4.1 (2) | 4.0 (1) | 5.1 (1) |
| FE ROM (Deg) | TEA | 7 | 49.1 ± 21.6 | 34.0 (11) | 66.5 (16) | 63.9 (18) | 57.0 (14) | 51.2 (23) | 36.3 (9) | 23.4 (20) | 60.9 (17) |
|  | Control | 18 | 62.2 ± 25 | 38.2 (12) | 85.6 (12) | 86.2 (19) | 71.2 (10) | 68.4 (13) | 39.4 (10) | 30.0 (11) | 78.3 (17) |
| PS ROM (Deg) | TEA | 7 | 58.9 ± 36.4 | 91.5 (40) | 52.1 (16) | 43.4 (20) | 25 (11) | 33.6 (21) | 91.7 (37) | 41.6 (20) | 92.3 (30) |
|  | Control | 18 | 57.4 ± 44.4 | 96.4 (43) | 38.4 (26) | 41.7 (23) | 44.1 (52) | 27.2 (23) | 67.8 (47) | 39.4 (24) | 104.5 (39) |
| FE Moment (Nm) | TEA | 6 | 6.8 ± 2.9 | 9.3 (4) | 10.1 (4) | 5.7 (4) | 6.6 (1) | 4.4 (1) | 4.6 (1) | 6.9 (1.4) | 7.2 (1.3) |
|  | Control | 17 | 8.0 ± 4.6 | 9.7 (2) | 9.8 (3) | 14.5 (9) | 7.0 (2) | 4.2 (1) | 4.2 (2) | 7.6 (2) | 7.3 (2) |
| VV Moment (Nm) | TEA | 6 | 7.4 ± 4.0 | 14.2 (2) | 12.8 (3) | 6.0 (3) | 6.3 (2) | 3.6 (1) | 4.7 (1) | 4.9 (2) | 8.1 (1) |
|  | Control | 17 | 7.9 ± 5.6 | 13.6 (4) | 11.5 (3) | 14.3 (9) | 5.5 (2) | 2.7 (1) | 4.4 (2) | 4.8 (1) | 7.2 (2) |
| Power (Watt) | TEA | 6 | 4.6 ± 4.7 | 5.2 (5) | 6.8 (5) | 3.7 (3) | 5.0 (6) | 2.9 (4) | 3.2 (2) | 2.7 (2) | 7.9(8) |
|  | Control | 17 | 7.0 ± 9.0 | 7.3 (5) | 9.2 (7) | 20.1 (17) | 5.7 (4) | 2.8 (3) | 3.0 (3) | 3.5 (5) | 5.1 (5) |
| Work - (Joule) | TEA | 6 | -2.1 ± 2.4 | 1.6 (1) | 1.7 (2) | 1.8 (2) | 2.8 (3) | 1.9 (2) | 1.2 (1) | 1.1 (1) | 4.2 (4) |
|  | Control | 17 | -1.9 ± 2.0 | 1.4 (1) | 1.3 (2) | 3.6 (3) | 2.3 (3) | 1.6 (1) | 0.7 (0.5) | 1.0 (1) | 3.3 (2) |
| Work + (Joule) | TEA | 6 | 2.9 ± 3.3 | 2.9 (1) | 6.8 (5) | 2.3 (3) | 2.7 (2) | 2.0 (2) | 1.4 (1) | 1.3 (1) | 4.6 (4) |
|  | Control | 17 | 3.7 ± 4.4 | 4.4 (3) | 8.4 (6) | 7.6 (7) | 2.7 (2) | 1.5 (1) | 1.0 (1) | 1.4 (2) | 2.8 (2) |
